# Announcing a New Journal Section: Cord Blood

**DOI:** 10.1002/sctm.17-0077

**Published:** 2017-04-29

**Authors:** Joanne Kurtzberg, Anthony Atala

This issue of *STEM CELLS Translational Medicine* marks an important milestone in the journal's mission to advance the clinical use of stem cells. Through a partnership with the Cord Blood Association, SCTM now includes a new section to showcase the newest and highest quality translational and clinical applications of cord blood and cord tissue‐based therapies.

Areas the new section will focus on include cord blood and cord tissue‐based therapies, accessory cell populations, engineering and manufacturing of cord blood immune cells, modulation of endogenous cell and tissue repair, cord blood and cord tissue banking, malignant and non‐malignant diseases treated by cord blood transplantation and cord blood and cord tissue in regenerative medicine.

For readers not familiar with CBA (cb‐association.org.), it is an international, nonprofit organization that promotes public and private cord blood banking and the use of umbilical cord blood and related tissues for disease treatment and regenerative therapies. CBA members include public and private cord blood banks as well as individuals in and served by the cord blood community. These include cord blood bank personnel, research investigators, laboratory technicians, patients, donors, regulatory officials, vendors and health care providers such as transplant physicians, cell therapists, obstetricians, nurses, and midwives.

CBA is dedicated to promoting the work of the cord blood community, saving human lives, changing medicine and improving domestic and international policy. Its main priorities are advocacy, market expansion, quality products and service, education, and research and development.

Cord blood has been used as a source of donor blood stem cells in allogeneic hematopoietic stem cell transplantation (HSCT) for almost 30 years. Repositories of banked related and unrelated cord blood have been established around the world. Procedures for collecting, processing, testing, cryopreserving, storing, shipping, thawing, and administration have been developed and largely standardized. Unrelated donor cord blood units are listed on donor registries and available for use by patients in need of a donor for HSCT worldwide. Unrelated donor cord blood is the only hematopoietic stem cell source that is eligible for licensure by the FDA and there are currently seven licensed banks in the U.S.

The first cord blood transplant, using matched sibling cord blood, was performed in a child in 1988. Regulations were less robust in those days, allowing this promising new cell‐based therapy to be tested in the clinic with minimal preclinical data. Fortunately, this first transplant and subsequent ones were successful, allowing the field of cord blood transplantation to develop and thrive. Early on, it was shown that cord blood cells were more immunologically tolerant, allowing transplantation without full HLA matching, providing access to transplantation therapies for patients lacking fully matched related or unrelated donors.

Even in the mismatched setting, cord blood caused less graft‐versus‐host disease without losing leukemia fighting activities. Banked cord blood is rapidly available, without donor attrition, with more than two decades of long‐term stability in the cryopreserved state. Rapid access to banked cord blood donors makes it an ideal donor source for babies and young children with certain inherited metabolic diseases and congenital immunodeficiency syndromes identified through newborn screening programs. Cord blood has been limited by the number of cells available for dosing from a single unit, but recent advances in cell expansion have led to the development of technologies that expand both stem and progenitor cells of the hematopoietic lineages.

As illustrated by the article by Dawson and colleagues in the inaugural cord blood section of the Journal 1, cord blood can be safely administered to children with autism and may reduce symptomatology associated with increasingly common and often devastating disease. In these cases, non‐stem cell populations of cells in cord blood act through paracrine signaling to promote tissue repair and regeneration. Engraftment is not required and, accordingly, patients are not pretreated with any immunosuppressive or myeloablative therapies.

This alternative use of cord blood cells, as intelligent surrogates for drugs, has enormous potential to treat many other medical conditions that currently have unmet needs. It also demonstrates that a cell product or tissue containing multiple types of cells can have multiple major homologous activities, each with different specificities and therapeutic actions in different clinical applications.

While this initial report utilized banked autologous cord blood cells, it is extremely unlikely that every patient in need will have access to their own cord blood. Accordingly, the next applications with this promising cell‐based therapy should focus on the use of allogeneic cord blood cells. The worldwide inventory of banked related and unrelated cord blood will provide ample donors for patients in need.

We look forward to sharing equally exciting and promising reports in future issues of the journal and we thank you, our readers, for continuing to look to SCTM for the latest advances in the field.

## References

[1] Dawson G , Sun JM , Davlantis KS et al. Autologous cord blood infusions are safe and feasible in young children with autism spectrum disorder: Results of a single‐center phase I open‐label trial. Stem Cells Translational Medicine 2017;6:1332–1339. 28378499 10.1002/sctm.16-0474 PMC5442708

